# Hepatoprotective Role of Ethanolic Extract of *Vitex negundo* in Thioacetamide-Induced Liver Fibrosis in Male Rats

**DOI:** 10.1155/2013/739850

**Published:** 2013-05-19

**Authors:** Farkaad A. Kadir, Normadiah M. Kassim, Mahmood A. Abdulla, Wageeh A. Yehye

**Affiliations:** ^1^Department of Anatomy, Faculty of Medicine, University of Malaya, 50603 Kuala Lumpur, Federal Territory, Malaysia; ^2^Department of Biomedical Science, Faculty of Medicine, University of Malaya, 50603 Kuala Lumpur, Federal Territory, Malaysia; ^3^Nanotechnology & Catalysis Research Centre (NANOCAT), Block 3A, Institute of Postgraduate Studies Building, University of Malaya, 50603 Kuala Lumpur, Federal Territory, Malaysia

## Abstract

The hepatoprotective activity of ethanolic extract from the leaves of *Vitex negundo* (VN) was conducted against thioacetamide- (TAA-) induced hepatic injury in *Sprague Dawley* rats. The therapeutic effect of the extract was investigated on adult male rats. Rats were divided into seven groups: control, TAA, Silymarin (SY), and VN high dose and low dose groups. Rats were administered with VN extract at two different doses, 100 mg/kg and 300 mg/kg body weight. After 12 weeks, the rats administered with VN showed a significantly lower liver to body weight ratio. Their abnormal levels of biochemical parameters and liver malondialdehyde were restored closer to the normal levels and were comparable to the levels in animals treated with the standard drug, SY. Gross necropsy and histopathological examination further confirmed the results. Progression of liver fibrosis induced by TAA in rats was intervened by VN extract administration, and these effects were similar to those administered with SY. This is the first report on hepatoprotective effect of VN against TAA-induced liver fibrosis.

## 1. Introduction

Liver is a vital organ of metabolism and excretion in the body. It is involved in the biochemical conversions of varied administered substances which significantly increased the reactive oxygen species generation [1]. These liberated radicals can be produced by hepatotoxins, such as thioacetamide (TAA). Several investigations have approved that single dose of this hepatotoxic agent could produce centrilobular hepatic necrosis, and chronic administration led to cirrhosis [2]. 

Although the modern medicinal system has developed phenomenally, discovering a new drug for treating liver diseases is still a dream. Therefore, a number of therapeutic plants are used in the traditional system of medicine for the management of liver disorders. However, many of them have not been investigated for their effects. VN is one such medicinal plant credited with numeral curative qualities validated by modern science and used since ancient times. VN belongs to a family of Verbenaceae and commonly called five-leaved chaste tree [3]. It is mainly distributed in tropical to temperate regions, especially in India [4], which is traditionally used by the native medical practitioners for the treatment of various ailments, including stomach-ache, disease of the eye, inflammation, enlargement of spleen, bronchitis, asthma, and painful teething in children [5]. 

The leaves are aromatic, tonic, and vermifuge [6]; the juice from the leaves was used for the treatment of ulcers and swelling of joints [7]. Preliminary phytochemical screening of the extract and literature survey shows the presence of alkaloid, flavonoids-like flavones, luteolin-7-glucoside, casticin, iridoid, glycosides, an essential oil, and other constituents like vitamin C, carotene, glucononital, benzoic acid, *β*-sitosterol,s and glycoside [3]. The literature reviews reveal that the plant VN possesses analgesic and antinociceptive activity [8], hepatoprotective activities against antitubercular drugs [3], CCl_4_ [9], and ibuprofen via inhibition of lipid peroxidation [10].

TAA is a very effective, reliable, and satisfactory model in producing liver cirrhosis in laboratory rodents. Various investigators have used different methods of TAA administration in experimental animals for producing fibrosis and cirrhosis, such as intraperitoneal or subcutaneous administration, mixing the toxin with the diet or in drinking water [11–13]. 

 Interestingly, a very recent patent was effectively produced using a new composition of treatment and prevention of liver disease based on plants' extracts [14]. Accordingly, this work was designed to determine the hepatoprotective effect of VN against TAA-induced liver fibrosis in male *Sprague Dawley* rats.

## 2. Materials and Methods

### 2.1. Collection and Preparation of Plant Extract

Fresh leaves of VN plant were obtained from Kampung Baru, Sungai Ara, Penang, Malaysia. The plant was identified, and the voucher specimen number (KLU 34968) was deposited in University of Malaya (Department of Pharmacy). The plant was dried, grounded to fine powder, and homogenized in 95% ethanol at a ratio of 1 : 10 of plant to ethanol. The mixture was left to soak for four days at 25°C with occasional shaking and stirring. Subsequently, the mixture was filtered using a filter paper, and the filtrate was concentrated in a reduced pressure at 45°C to obtain a dark gummy-green extract. The extract was then dissolved in Tween 20 (10%, w/v) and administered orally to rats at dose 100 and 300 mg/kg body weight.

### 2.2. Preparation of Thioacetamide (TAA)

TAA (from Sigma-Aldrich, Switzerland) and all other chemicals used were of analytical grade and purchased mostly from Sigma-Aldrich and Fisher. TAA stock solution was prepared by dissolving 30 mg pure TAA which is in crystal form in 100 mL distilled water (0.03% w/v) until all the crystals were dissolved. The solution was given to the rats as their daily drinking water. Constant exposure of a rat to this amount of TAA induces changes in its liver pathology for both biochemical and morphological aspects comparable to that of human liver cirrhosis [11]. 

### 2.3. Preparation of Silymarin (SY)

SY with 80% purity, (International Laboratory, USA) as a standard drug, was dissolved in Tween 20 (10% w/v) and orally administered to rats at a dose of 50 mg/kg body weight [15].

### 2.4. Animals

Fourty-two adult healthy male *Sprague Dawley *(SD) rats weighing 180–200 g were obtained from Animal House Unit, Faculty of Medicine, University of Malaya, Malaysia. The experimental protocol was approved by Animal Ethics Committee with an ethical number: ANA/18/05/2012/FAAK. The animals were kept in wire-bottomed cages at 25 ± 3°C, 50–60% humidity, and a 12 h light-dark cycle for at least a week before the start of the experiment and were maintained under standard housing conditions with free access to standard diet and water ad libitum. Throughout the experiment, all the criteria of taking care of animals prepared by the National Academy of Sciences and outlined in the “Guide for the Care and Use of laboratory Animals” were applied.

The animals were randomly divided into seven groups of six rats each and treated as follows. Group 1: normal control, rats were administered orally with 10% Tween 20 (5 mL/kg) daily. Group 2: TAA group, rats were given 0.03% TAA with their drinking water daily. Group 3: SY group, rats were given SY 50 mg/kg orally + 0.03% TAA daily (SY is a well-known standard drug with hepatoprotective activity). Group 4: low dose VN group, rats were given VN 100 mg/kg orally. Group 5: low dose VN + TAA group, rats were given VN 100 mg/kg orally + 0.03% TAA daily. Group 6: high dose VN group, rats were given VN 300 mg/kg orally. Group 7: high dose VN + TAA group, rats were given VN 300 mg/kg orally + 0.03% TAA daily. 

The experiment was carried out for a total of 12 weeks. The body weights of the animals were recorded weekly beginning from day 0 and throughout to the end of the experiment. At the end of the 12th week, the rats were sacrificed 24 hours after the last treatment. The rats were fasted overnight and then anaesthetized by intramuscular injection of 50 mg/kg ketamine mixed with xylazine 5 mg/kg. Blood samples were collected and centrifuged; serum was separated for assay of the biomarkers.

#### 2.4.1. Measuring the Body Weight and Relative Liver Weight

The liver and spleen of the rats were isolated, dissected, and then rinsed in normal saline. Following this, the organs were blotted with filter paper and weighed. Gross examination was conducted to check for gross abnormalities of the organs and photographs were taken. The liver and spleen indices were calculated as the percentage of the body weight. Each liver was excised into two pieces. The right lobe was immersed in isotonic 10% buffered formalin fixative for histological assessment while the left lobe was rinsed using cold physiological saline and then homogenized with cool phosphate buffer saline for MDA assay.

#### 2.4.2. Biochemical Analysis

Blood was withdrawn through the jugular vein and collected into a plain tube containing activated gel for liver function tests. The sample was allowed to clot, before being centrifuged to separate the serum. Serum samples were sent to the Clinical Diagnosis Laboratories, University of Malaya Medical Centre to determine the levels of alanine aminotransferase (ALT), aspartate aminotransferase (AST), alkaline phosphatase (AP) levels, bilirubin, and total serum proteins including albumin and lipid profiles using standard automated techniques according to the procedures described by the manufacturers.

#### 2.4.3. Histopathological Evaluation

The fixed tissues were processed by automated tissue processing machine (Leica, Germany). Tissues were embedded in paraffin wax by conventional methods. Sections of 5 **μ**m in thickness were prepared and then stained with Masson's trichrome stain. Afterwards, the sections were observed under the light microscope for histopathological changes, and representative areas were photomicrographed.

#### 2.4.4. Estimation of Malondialdehyde (MDA) in Liver Tissue

Liver samples were rinsed three times using cold physiological saline (0.9% NaCl). Each piece of liver was then homogenized with 10 mL volume of cool phosphate buffer saline (10% w/v) in a glass-Teflon homogenizer. The homogenate was centrifuged at 3500 rpm in an automatic high-speed cold centrifuge for 10 minutes at 4°C. The supernatant was collected for estimation of lipid peroxidation product, MDA content, by using a commercially available kit (Cayman Chemical Company, USA). MDA level in liver tissue was determined based on the reaction of MDA with the thiobarbituric acid method [16] forming an MDA-TBA_2 _that absorbs strongly at 532 nm.

### 2.5. Quantitative Estimation of Total Phenolic Content (TPC)

Total phenolic content of VN was carried out using Folin-Ciocalteu reagent according to the reported method [17]. The samples were inserted into different test tubes and mixed thoroughly with 5 mL Folin-Ciocalteu reagent. After 5 minutes, 4 mL of sodium carbonate (7.5% Na_2_CO_3_) was added and allowed to react for two hours at room temperature. The absorbance was measured at 765 nm using microplate reader spectrophotometers. Quercetin was used as a reference compound to produce a standard curve, and the results were expressed as mg of quercetin equivalents to gm VN extract. This assay was carried out in triplicate.

### 2.6. Total Flavonoids Determination (TFC)

Total flavonoid content of VN sample was determined by aluminium chloride colorimetric method by Chang et al., 2002 [18]. Briefly, 0.5 mL of extract solution (1 mg/1 mL) was added to a separate test tube and mixed with 1.5 mL of 95% ethanol, 0.1 mL of 1 M of potassium acetate, 0.1 mL of aluminium chloride, and 2.8 mL of distilled water. Subsequently, the mixture was incubated for 30 minutes at room temperature. The absorbance readings were taken spectrophotometrically at 415 nm. A yellow color indicated the presence of flavonoids. Total flavonoid content was expressed as milligram of quercetin equivalent to gram of dried plant material.

### 2.7. Statistical Analysis

The results were presented as mean ± standard error mean. The one-way ANOVA test with post hoc test using Bonferroni multiple comparisons in the PASW program (version 18) for Windows (SPSS Inc., Chicago, IL, USA) was used to analyse the data, with *P* < 0.05 being considered as the limit of significance.

## 3. Results

### 3.1. Gross Morphology

**Figure 1:**
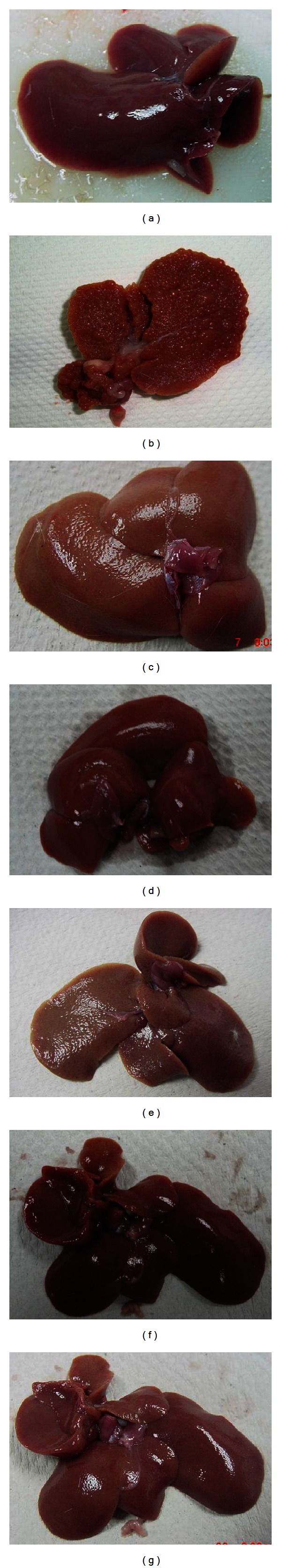
Photographs showing the macroscopic appearances of livers from different experimental groups: (a) control group—showing regular smooth surface, (b) TAA group (hepatotoxic group)—showing shrinkage of the liver with multiple cirrhotic nodules on the whole surface of the liver, (c) SY + TAA group—showing smooth surface, (d) VN 100 group—showing liver with a smooth surface, (e) VN 100 + TAA group—showing liver with a nearly smooth surface, (f) VN 300 group—showing liver with a smooth surface, and (g) VN 300 + TAA group—showing liver with a smooth surface.

Grossly, the livers of the TAA group (G2) were congested, and the liver surfaces showed many spots of nodules, firm in consistency and relatively harder in comparison with the normal gross features of the livers from the control (G1), VN 100 (G4), and VN 300 (G6) groups. In contrary, the livers from VN 100 + TAA (G3), VN 300 + TAA (G5), and SY + TAA (G7) treated group, were with much fewer nodular spots, softer in consistency, and with a comparatively normal liver size than the TAA group (Figure 1).

### 3.2. Body, Liver, and Spleen Weight

In the present investigation, there was a statistically significant reduction of body weight in rats administered with TAA compared to the normal group and VN 100 and VN 300 groups. Although there was an increase in body weight in VN 300 + TAA, VN 100 + TAA, and SY + TAA, but still there was no significant change in comparison to TAA group (Table 2). However, there was a significant change in the percentage of the mean body weight for all treated groups in comparison to TAA group (data not shown). Concurrently, the liver to body weight ratio showed a corresponding significant difference between the treated groups compared to TAA group. Furthermore, there were significantly higher spleen weight and spleen index for TAA group in comparison to all other experimental groups (Table 2).

### 3.3. Biochemical and Antioxidant Parameters

**Figure 2:**
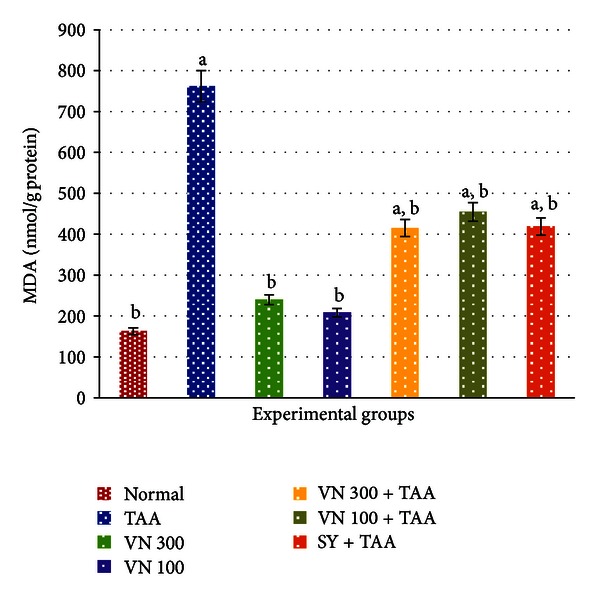
Effect of VN extract on MDA level in all experimental groups. The data were stated as mean ± SEM. Means with different superscripts are significantly different. ^a^ *P < *0.05 versus normal control group and ^b^ *P < *0.05 versus TAA control group. SY stands for Silymarin (standard hepatoprotective drug). The experiment was carried out in triplicate.

Administration of long-term TAA had caused a significant increase in biochemical markers including ALT, AST, ALP, bilirubin, and MDA levels with a decrease in total protein and albumin compared to the normal- and VN-treated groups, which indicated acute liver damage. Low and high dose of VN extract and SY significantly reduced the level of liver enzymes ALT, AST, ALP, bilirubin levels (Table 1), and MDA level (Figure 2), in addition to significant increase in total protein and albumin levels compared with the TAA group (Table 1). The toxic effect of TAA was affectively alleviated by the administration of VN extract at 300 mg/kg body weight compared with its marginal effect at 100 mg/kg body weight. Although treatment with VN extract did not reduce the levels of liver enzymes, bilirubin, and MDA to those of the normal group, VN extract at 300 mg/kg body weight showed a similar potent effect of SY in protecting the rat against TAA-induced liver damage, as evidenced by the reduction of all enzyme levels of AST, ALT, and ALP and increase in total protein and albumin levels compared with the TAA group (Table 1).

On the other hand, the lipid profile has been significantly elevated in TAA group, and the treated groups with the low and high dose of VN resulted in significant reduction in lipids including total cholesterol, triglyceride, and low-density lipoprotein (Table 3).

### 3.4. Total Phenolic and Flavonoids Contents

The ethanolic extract of VN contains appreciable amounts of phenolic compounds as phenolic and flavonoids with values 249.00 ± 0.002 mg and 120.90 ± 0.003 mg quercetin/g extract, respectively. 

### 3.5. Histological Findings

Livers from the normal control group showed normal liver architecture with normal hepatocytes and portal lobules, while in TAA-treated groups, the livers showed loss of normal architecture with the presence of regenerating nodules separated by prominent fibrous septa extending from the central vein. There was abnormal appearance of the portal tracts apart from twinning of cell plates due to regenerating activity of the hepatocytes, as well as presence of some inflammatory cells. In VN-treated animals, liver sections showed relatively mild inflammation and mild cytoplasmic vacuolation, while most areas showed no visible changes. Histopathological examination also showed good recovery of TAA-induced fibrosis by ethanolic extracts of VN as compared to SY.

**Figure 3:**
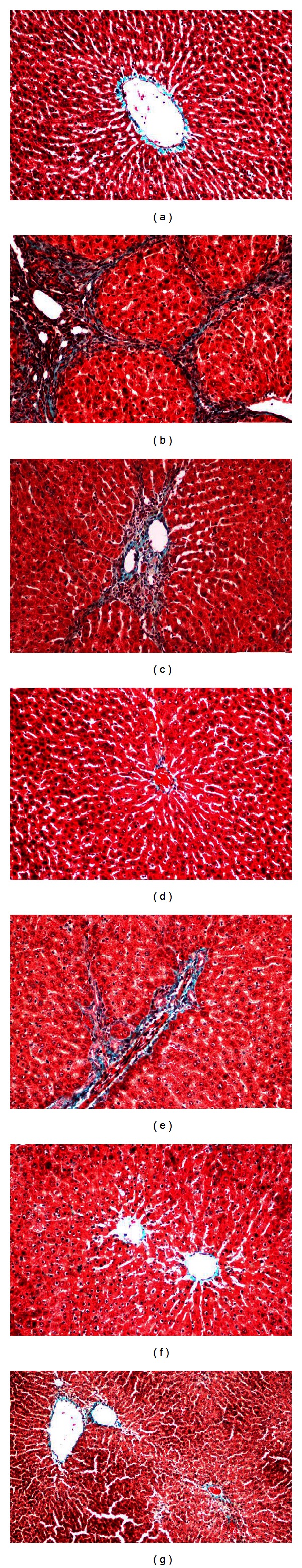
Photomicrographs showing the histopathological images of livers from different experimental groups: (a) control group—showing normal liver architecture, (b) TAA group (hepatotoxic group)—showing proliferation of bile duct and thick fibrous septa, (c) SY group—showing mild fibrous septa, (d) VN 100 group—showing normal liver architecture, (e) VN 100 + TAA group—showing multiple nodules with moderate fibrous septa and fewer cirrhotic nodules, (f) VN 300 group—showing normal liver architecture, and (g) VN 300 + TAA—showing mild fibrous septa. Masson's trichrome stain. Magnification ×200.

Animals treated with low dose VN showed regeneration of hepatocytes surrounded by septa of fibrous tissue, while those treated with the higher dose of the plant extract showed remarkable histological regeneration. The latter showed nearly normal patterns with an increased area of normal liver parenchyma and a reduced development of fibrous septa and lymphocyte infiltration (Figure 3).

Each liver section was subjectively scored by two blinded experienced observers, including an anatomist and histopathologist for evidence of fibrosis, fatty change, architectural distortion, and regenerative nodules. The extent of bile duct proliferation and fibrosis by using Masson's trichrome stain were duly graded by a semiquantitative method on a scale between zero and six [19]. Stage 0 values were indicative of normal hepatic architecture in which there was no evidence of bile duct proliferation, inflammation, and fibrosis, whereas stage 6 reflected a gross disturbance of liver architecture indicated by marked proliferation of the bile ducts with severe inflammation and a very conspicuous/prominent fibrotic response (Table 4). Each sample was viewed under ×200 magnification. The degree of fibrosis was expressed as the mean of 10 different fields in each slide [20]. 

## 4. Discussion

One of the major functions of the liver is detoxification of xenobiotics and toxin. TAA is a potent hepatotoxic agent that is metabolized by Cytochrome 450 enzyme present in liver and is converted by oxidative chains to toxic substances called TAAS-oxide and TAAS-dioxide [21]. TAA is known to induce centrilobular hepatic necrosis, liver cirrhosis, hepatocellular carcinoma, and bile duct proliferation [22]. TAA-induced liver fibrosis is caused by free radical-mediated lipid peroxidation [23]. 

In chronic TAA intoxication, substantial liver fibrosis and prominent regenerative nodule development are associated with portal hypertension and hyperdynamic circulation characteristic of liver cirrhosis [24]. This is evidenced by the histopathological analysis and biochemical parameters (ALT, AST, AP, and bilirubin) in plasma. However, the mechanism of oxidative stress in TAA hepatotoxicity is still unclear. 

In recent years, many studies have shown that various types of natural products have a wide range in biochemical, pharmacological, and physiological effects due to the properties of their constituents [20, 25, 26]. In particular, they contain many types of polyhydroxy phenol compounds, which can function as natural antioxidants in humans and animals [27, 28]. Accordingly, the present study was undertaken to assess the effect of VN in rat model of chronic liver disease in order to confirm that this plant does indeed have a preventive benefit in liver disease.

It has been reported that administration of TAA in drinking water for twelve weeks induces liver fibrosis [29]. This result has been proven by the significantly different levels of biochemical markers between the TAA control and another plant-treated groups. In the present study, animals treated with 300 mg/kg of VN ethanolic extract exhibited a hepatoprotective effect comparable to those treated with 50 mg/kg of SY in TAA-induced liver injury. Treatment with VN extract at 100 mg/kg and 300 mg/kg body weight restored the biochemical parameters (ALT, AST, AP, and bilirubin) towards normal (Table 1). Furthermore, TAA group strongly showed significant elevation of serum cholesterol, triglyceride and LDL levels in comparison to VN-and SY-treated groups (Table 3). This had most probably occurred in chronic liver diseases as a result of deficiency of a functional LDL receptor which renders the inability of the liver to clear LDL cholesterol from the blood stream [30]. 

Since the metabolism of proteins, synthesis of amino acid and urea occurs in the liver; we anticipated alterations in the concentrations and characteristics of these materials in liver disease. Hence, the levels of total serum albumin concentration in TAA groups concurrently treated with low and high dose of VN and SY were significantly higher in comparison to untreated TAA group (Table 1). Presumably, these alterations are more marked in chronic conditions, as in our case of cirrhosis. Reduced level of albumin usually follows the severity of the condition, and it is a reliable index of the prognosis [31]. Generally, the continuation of hypoalbuminaemia indicates a progress or a change to a chronic state. The significant improvement at the level of biochemical parameters is indicative of the beneficial effects of the selected plant extract in protecting the liver.

From our data, TAA group showed a marked reduction in body weight with a significant increase in liver and spleen weights compared to SY- and VN-treated groups (Table 2). This reduction in body weight could strongly be attributed to the toxic effect of TAA throughout the period of the experiment [11]. This is considered to be the most reliable and consistent symptoms of toxicity among the experimental animals [32]. Accordingly, the increase in spleen weight was most probably due to the consequence of elevated portal venous pressure and subsequent engorgement of the organ [33]. Our findings showed no significant increase in the body weight of animals concurrently treated with VN extract when compared to TAA group. However, the normal increase in body weight of rats treated only with VN extracts indicates the overwhelming safety of this plant. 

From the histopathological findings, treatment with VN extract showed recovery of liver structure in TAA-induced liver cirrhosis in rats. Indeed, there was remarkable reduction in the extent of fibrosis, which is probably due to reduced amount of stellate cells infiltration in rats treated with the plant extract compared to TAA group. TAA-treated rat liver showed fatty degeneration and necrosis. These effects were nearly normalized in the histoarchitecture of livers in the VN-treated rats, especially in the high dose (VN 300 mg/kg) group where the nodules of hepatocytes were separated only by thin fibrous septa, surrounding the degenerating hepatocytes induced by TAA (Figure 3). 

As the portal tracts are the sites at which circulating lymphoid cells first gain access to the liver [34], hence, portal inflammation is common in many liver diseases. In the present study, many inflammatory cells and proliferations of the bile ducts at the portal area were seen mainly in TAA group. In contrary, it looks less deteriorated in SY- and low dose VN-treated groups while almost normalized in high dose VN-treated group (Figure 3). Therefore, it is strongly believed that ethanolic extract of VN has a hepatoprotective effect against TAA-induced liver damage in rat. Thus, our observation supports the earlier finding established by Mahalakshmi et al., 2010 [10]. 

A previous preliminary acute toxicity study conducted for this plant revealed the nontoxic nature of VN on normal rats, and the LD_50_ dose recorded was 7.58 g/kg body weight. In addition, there were no histomorphological changes in liver, stomach, heart, and lung in any of the doses of the extract studied. This is consistent with our findings on the levels of the biochemical parameters (Table 1) and lipid profiles (Table 3), together with the histological findings (Figure 3) for rat livers in VN 100 and VN 300 groups. 

Antioxidants are essential to destroy the free radicals that exist in our body [35], and flavonoids have been identified as powerful antioxidants [36]; moreover, several studies have demonstrated the beneficial effects of antioxidant in protecting the liver against TAA-induced injury [35]. Concurrently, the effect of SY on liver cells suggests that the hepatoprotective action is due to the presence of flavonoids and phenols and their regenerative ability and its antioxidant effects [37, 38].

Several phytochemical studies revealed the presence of volatile oils, lignans, flavonoids-like flavones, luteolin-7-glucoside, glycosides, and phenols in VN [4]. Besides, VN ethanolic extract possesses radical scavenging activity probably due to its higher concentration of flavonoids and phenols [39]. These findings are in agreement with our results. The phenolic components most frequently present in ethanol extract of VN include negundoside, agnuside, vitegnoside, 7,8 dimethyl herbacetin 3-rhamnoside, 5,3′-dihydroxy—7,8,4′-trimethoxy flavanone, 5-hydroxy-3,6,7,3′,4′-pentamethoxy flavone, 5,7 dihydroxy-6,4′ dimethoxy flavanone, and 5 hydroxy-7,4′ dimethoxy flavones. Among these components, negundoside is the most active phenol as an antioxidant. It was found to protect the liver against CCl_4_-induced liver toxicity and oxidative stress. The suggested mechanism of protection was due to decreased production of ROS and lipid peroxidation. Other phenolic components such as agnuside, vitegnoside, and flavonoids are also present in the plants as natural antioxidants [40]. This is evident from the photomicrographs on livers (Figure 3) and the decrease in MDA level (Figure 2) as well as the reduced lipid peroxidation in VN*-*treated groups. 

## 5. Conclusions

All the data obtained from this study showed strong preliminary evidence that VN ethanolic extract has hepatoprotective effects against liver toxicity induced by TAA as proven by macroscopical, microscopical, and biochemical analyses. The effects of VN are comparable to that of SY, the standard hepatoprotective drug. Accordingly, VN extract could be used as an effective herbal product for the prevention of chemical-induced hepatic damage. It is believed to be due to its flavonoid content and its antioxidant activity. In the near future, a further study is warranted to isolate, characterize and screen the active components of VN that have the hepatoprotective activity. 

## Figures and Tables

**Table 1:** Body weight and liver and spleen weights of the rats in different groups.

Group	Body weight (g)	Liver weight (g)	Liver index (%)	Spleen weight (g)	Spleen index (%)
Normal	219.8 ± 29.88^b^	6.78 ± 0.71^b^	3.13 ± 0.51^b^	0.495 ± 0.08^b^	0.227 ± 0.03^b^
TAA	177.5 ± 7.1^a^	8.27 ± 0.99^a^	4.66 ± 0.57^a^	0.863 ± 0.13^a^	0.488 ± 0.08^a^
VN 300	226.16 ± 22.62^b^	6.78 ± 0.69^b^	3.46 ± 0.85^b^	0.647 ± 0.12^b^	0.287 ± 0.05^b^
VN 100	221.15 ± 15.91^b^	6.69 ± 0.55^b^	3.04 ± 0.38^b^	0.643 ± 0.68^b^	0.24 ± 0.12^b^
VN 300 + TAA	204.3 ± 10.70	6.9 ± 0.67^b^	3.39 ± 0.40^b^	0.641 ± 0.07^b^	0.313 ± 0.03^b^
VN 100 + TAA	199.3 ± 15.98	7.2 ± 0.27	3.64 ± 0.39^b^	0.588 ± 0.10^b^	0.295 ± 0.06^b^
SY + TAA	210.16 ± 12.05	7.03 ± 0.40	3.34 ± 0.09^b^	0.602 ± 0.15^b^	0.23 ± 0.031^b^

The data were stated as mean *±* SEM. Means with different superscripts are significantly different. ^a^ *P* < 0.05 versus normal control group and ^b^ *P* < 0.05 versus TAA group. SY stands for Silymarin (standard hepatoprotective drug).

**Table 2:** Effect of TAA, SY, and VN ethanolic extract on liver function test.

Group	ALT (IU/L)	AST (IU/L)	AP (IU/L)	Bilirubin (umol/L)	Total protein (mg/dL)	Albumin (g/L)
Normal	43.66 ± 4.9^b^	23.66 ± 3.1^b^	87.66 ± 25.08^b^	4.83 ± 2.6^b^	70.167 ± 2.9	41 ± 1.5^b^
TAA	89.83 ± 17.9^a^	311.33 ± 50.05^a^	223.8 ± 77.5^a^	19.83 ± 6.4^a^	69 ± 2.5	18.33 ± 4.8^a^
VN 300	50.16 ± 8.7^b^	31.16 ± 5.60^b^	81.66 ± 48.3^b^	5.66 ± 1.9^b^	74.167 ± 4.16	39 ± 3.2^b^
VN 100	55.33 ± 8.1^b^	32.33 ± 5.64^b^	101 ± 42.23^b^	7 ± 1.5^b^	78.3 ± 3.72^a,b^	38.83 ± 4.07^b^
VN 300 + TAA	60 ± 4.8^b^	128.83 ± 60.0^b,a^	107 ± 8.5^b^	9.33 ± 1.0^b^	72 ± 2.6	35.6 ± 4.8^b^
VN 100 + TAA	62 ± 4.1^a,b^	152.5 ± 47.0^b,a^	136 ± 39.6^b^	9.16 ± 1.4^b^	72.83 ± 3.31	31.83 ± 10.6^b^
SY + TAA	59.16 ± 4.5^b^	154.33 ± 62.7^b,a^	139 ± 37.5^b^	8 ± 1.6^b^	75.167 ± 4.2	36.83 ± 5.6^b^

The data were stated as mean *±* SEM. Means with different superscripts are significantly different. ^a^ *P* < 0.05 versus normal control group and ^b^ *P* < 0.05 versus TAA control group. SY stands for Silymarin (standard hepatoprotective drug).

**Table 3:** Effect of TAA, SY, and VN ethanolic extract on lipid profile.

Group	Total cholesterol (mmol/L)	Triglyceride (mmol/L)	LDL (mmol/L)
Normal	2.30 ± 0.38^b^	0.56 ± 0.22^b^	0.05 ± 0.72^b^
TAA	6.17 ± 0.70^a^	2.75 ± 0.52^a^	2.76 ± 0.88^a^
VN 300	2.00 ± 1.07^b^	0.53 ± 0.15^b^	0.04 ± 0.20^b^
VN 100	2.16 ± 0.65^b^	0.48 ± 0.13^b^	0.08 ± 0.55^b^
VN 300 + TAA	2.55 ± 0.55^b^	0.83 ± 0.55^b^	0.72 ± 0.51^b^
VN 100 + TAA	3.31 ± 0.39^b^	1.41 ± 0.45^b,a^	0.72 ± 0.96^b^
SY + TAA	2.41 ± 1.60^b^	0.81 ± 0.53^b^	0.96 ± 0.20^b^

The data were stated as mean *±* SEM. Means with different superscripts are significantly different. ^a^ *P* < 0.05 versus normal control group and ^b^ *P* < 0.05 versus TAA control group. SY stands for Silymarin (standard hepatoprotective drug).

**Table 4:** Stages of liver cirrhosis in all groups of rats after 12 weeks of treatment.

				Pathological staging					
Group	*N*	0	I	II	III	IV	V	VI	Average of stages
Normal	6	6	0	0	0	0	0	0	0.0^b^
TAA	6	0	0	0	0	2	2	2	5.00 ± 0.89^a^
VN 300 + TAA	6	2	4	0	0	0	0	0	0.66 ± 0.51^b^
VN 100 + TAA	6	1	2	3	1	0	0	0	1.33 ± 0.81^b,a^
SY + TAA	6	0	5	1	0	0	0	0	1.16 ± 0.408^b,a^

The data were expressed as mean ± SEM. Means with different superscripts are significantly different. ^a^ *P* < 0.05 versus normal control group and ^b^ *P* < 0.05 versus TAA control group. SY stands for Silymarin (standard hepatoprotective drug).
