# Corrigendum to “Hepatoprotective Role of Ethanolic Extract of* Vitex negundo* in Thioacetamide-Induced Liver Fibrosis in Male Rats”

**DOI:** 10.1155/2018/8464628

**Published:** 2018-11-05

**Authors:** Farkaad A. Kadir, Normadiah M. Kassim, Mahmood A. Abdulla, Wageeh A. Yehye

**Affiliations:** ^1^Department of Anatomy, Faculty of Medicine, University of Malaya, 50603 Kuala Lumpur, Federal Territory, Malaysia; ^2^Department of Biomedical Science, Faculty of Medicine, University of Malaya, 50603 Kuala Lumpur, Federal Territory, Malaysia; ^3^Nanotechnology & Catalysis Research Centre (NANOCAT), Block 3A, Institute of Postgraduate Studies Building, University of Malaya, 50603 Kuala Lumpur, Federal Territory, Malaysia

In the article titled “Hepatoprotective Role of Ethanolic Extract of Vitex negundo in Thioacetamide-Induced Liver Fibrosis in Male Rats” [1], there was figure duplication in Figure 1, which showed the appearance of livers. Figure 1(b) is the same as Figure 1, TAA, in another article by the same group, Amin et al. [2], and Figures 1(f) and 1(g) in [1] are the same.

An institutional investigation by the University of Malaya found there was no system to index and file data and images to avoid mislabeling and mishandling, which led to errors and duplication of research data. The authors did not thoroughly check the manuscript before submission.

Figures 1(b) and 1(f) were uploaded at revision after a reviewer asked for higher quality images. These images were selected in error by the authors. Moreover, Figures 1(c), 1(f), and 1(g) are replaced by new images as the authors informed us that they had taken images at the end of the experiment. Accordingly, they selected new images to replace the previous images in these panels. The corrected Figure 1 is shown below.

## Figures and Tables

**Figure 1:**
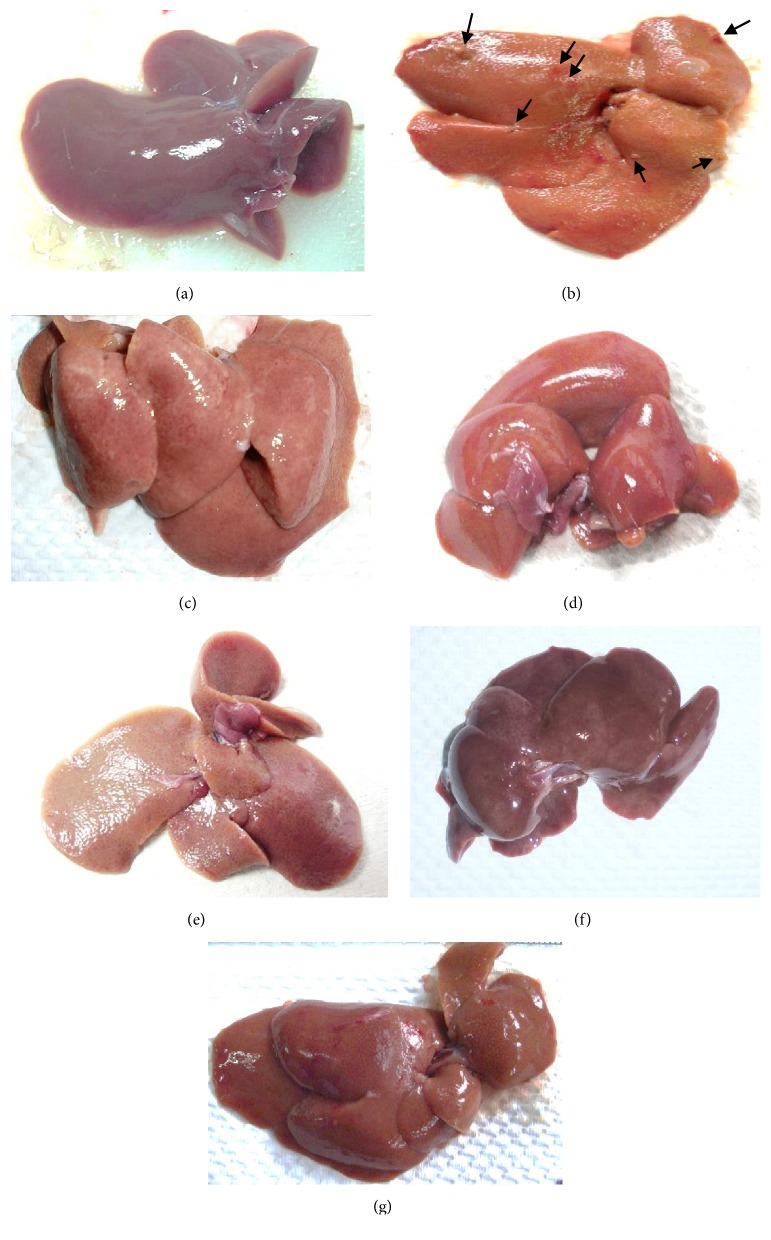
Photographs showing the macroscopic appearances of livers from different experimental groups: (a) control group—showing regular smooth surface, (b) TAA group (hepatotoxic group)—showing rough surface with micronodular distribution (black arrows), (c) SY + TAA group—showing smooth surface, (d) VN 100 group—showing liver with a smooth surface, (e) VN 100 + TAA group—showing liver with a nearly smooth surface, (f) VN 300 group—showing liver with a smooth surface, and (g) VN 300 + TAA group—showing liver with a smooth surface.

## References

[1] Kadir F. A., Kassim N. M., Abdulla M. A., Yehye W. A. (2013). Hepatoprotective role of ethanolic extract of *Vitex negundo* in thioacetamide-induced liver fibrosis in male rats. *Evidence-Based Complementary and Alternative Medicine*.

[2] Amin Z. A., Bilgen M., Alshawsh M. A., Ali H. M., Hadi A. H. A., Abdulla M. A. (2012). Protective role of *Phyllanthus niruri* extract against thioacetamide-induced liver cirrhosis in rat model. *Evidence-Based Complementary and Alternative Medicine*.

